# Risk Factors and Predictive Score Model for Early Recurrence After Curative Surgery in Patients With Poorly Differentiated Gastrointestinal Neuroendocrine Neoplasms

**DOI:** 10.3389/fsurg.2021.703138

**Published:** 2021-09-16

**Authors:** Chengguo Li, Peng Zhang, Xiong Sun, Xin Tong, Xin Chen, Chong Li, Wenchang Yang, Weizhen Liu, Zheng Wang, Kaixiong Tao

**Affiliations:** Department of Gastrointestinal Surgery, Union Hospital, Tongji Medical College, Huazhong University of Science and Technology, Wuhan, China

**Keywords:** neuroendocrine neoplasms, gastrointestinal, early recurrence, predictive model, risk factors

## Abstract

**Purpose:** Studies on early recurrence in gastrointestinal neuroendocrine carcinoma (NEC) and mixed adenoneuroendocrine carcinoma (MANEC) are lacking and risk factors related to early recurrence are not clear. We evaluated risk factors for early recurrence in such patients and developed a predictive scoring model.

**Methods:** Patients undergoing curative surgery for GI-NEC or MANEC between January 2010 and January 2019 were included. Early recurrence was defined as recurrence within 12 months after surgery. Risk factors for early recurrence were identified using logistic regression.

**Results:** Of the 80 included patients, 27 developed early recurrence and 53 had no early recurrence. Independent risk factors associated with early recurrence included tumor location in the midgut/hindgut [odds ratio (OR) = 5.077, 95% confidence interval (CI) 1.058–24.352, *p* = 0.042], alkaline phosphatase (ALP) >80 (OR = 5.331, 95% CI 1.557–18.258, *p* = 0.008), and lymph node ratio (LNR) >0.25 (OR = 6.578, 95% CI 1.971–21.951, *p* = 0.002). Risk scores were assigned to tumor location (foregut, 0; midgut/hindgut, 1), ALP (≤80, 0; >80, 1), and LNR (≤0.25, 0; >0.25, 1). Patients with a high risk (score 2–3) for early recurrence had significantly shorter disease-free survival and overall survival than those with low- (score 0) and intermediate risks (score 1) (both *p* < 0.001). The novel scoring model had superior predictive efficiency for early recurrence over TNM staging (area under the curve 0.795 vs. 0.614, *p* = 0.003).

**Conclusion:** Tumor location, preoperative ALP, and LNR were independent factors associated with early recurrence after curative surgery for GI-NEC or MANEC. The risk scoring model developed based on these three factors shows superior predictive efficiency.

## Introduction

Neuroendocrine neoplasms (NENs), formerly known as “carcinoids,” are highly heterogeneous neoplasms originating from sensory and secretory neuroendocrine cells (1). The incidence of NENs has increased over the past few decades, from 1.09 per 100,000 individuals in 1973 to 6.98 per 100,000 in 2012 (2). NENs can occur in various locations of the body, such as the lung, pancreas, gastrointestinal tract, and thymus, with the gastrointestinal tract being the most common affected site (3). The 2010 World Health Organization (WHO) classification of tumors of the digestive system categorizes NENs according to the degree of tumor cell differentiation: well or moderately differentiated neuroendocrine tumor (NET), poorly differentiated neuroendocrine neoplasms (PDNEN) including neuroendocrine carcinoma (NEC) and mixed adenoneuroendocrine carcinoma (MANEC) (4).

Surgical resection remains the mainstay of treatment for patients with gastrointestinal PDNEN (GI- PDNEN) (5). Adjuvant systemic chemotherapy is often also required for patients with a high degree of malignancy. Previous studies have reported that the 1-year progression-free survival of GI-PDNEN patients varies from 52 to 58%, indicating that a considerable number of patients will develop early recurrence within 12 months after surgery, despite treatment initiation with various adjuvant chemotherapy regimens (6–8). Early recurrence is closely related to poor prognosis; therefore, early screening of GI-PDNEN patients who are at high risk of early recurrence is essential for their improved prognosis. However, thus far, the risk factors associated with early recurrence of GI-PDNEN are not clearly analyzed.

Therefore, we focus on the early recurrence of patients with GI-PDNEN and aimed to evaluate the associated risk factors. Furthermore, we also aimed to establish a prediction model for early recurrence of such patients, which may help clinicians to screen GI-PDNEN patients according to the risk of early recurrence and guide clinical treatment.

## Materials and Methods

### Patient Selection

This retrospective study has been approved by the Ethics Committee of Union Hospital, Tongji Medical College, Huazhong University of Science and Technology (NO. 2021-0181). Two hundred and sixty-one patients with primary gastrointestinal NENs diagnosed at Union Hospital, Tongji Medical College, Huazhong University of Science and Technology from January 2010 to January 2019 were included in this study. The exclusion criteria were as follows: (1) patients who did not receive complete resection of NENs (*n* = 32); (2) patients diagnosed with NET (*n* = 119); (3) patients with a history of other malignant neoplasms (*n* = 3); (4) patients who received preoperative radiotherapy and chemotherapy (*n* = 4); (5) patients lost to follow-up (*n* = 9); and (6) patients who died of other reasons other than GI-PDNEN within 12 months postoperation (*n* = 7; three died of cerebrovascular disease, two died of heart disease, one died of chronic obstructive pulmonary disease, and one died of a car accident); (7) patients with distant metastasis at first visit (*n* = 6). Informed consent was obtained from all patients included in this study.

### Pathological Diagnosis

GI-NEC and GI-MANEC were histopathologically defined according to the WHO 2010 classification (4). The sections contained typical morphological findings and neuroendocrine markers including chromogranin A and synaptophysin as observed on immunohistochemical staining. GI-NEC was poorly differentiated with mitotic count >20/10 high-power field (HPF) and/or Ki-67 index >20%. If the grade of Ki-67 index was not in agreement with the grade of the mitotic rate, the parameter with the highest grade was used for classification. GI-MANEC was referred to as a carcinoma with at least 30% of neuroendocrine or non-neuroendocrine neoplasms. The pathological TNM stage of GI-PDNEN was re-evaluated according to the 8th Edition of TNM Classification issued by the American Joint Committee on Cancer (AJCC) (9).

### Definitions and Data Collection

Recurrence was diagnosed according to radiologic findings or biopsies with suspicious lesions. Patients with early recurrence, defined as recurrence within 12 months after surgery, were included in the early recurrence group, while patients with recurrence after 12 months or no recurrence were included in the non-early recurrence group.

Preoperative complete blood counts and alkaline phosphatase (ALP) levels were measured within seven days before surgery. The platelet-to-lymphocyte ratio (PLR) was defined as the ratio of the number of platelets to the number of lymphocytes. The neutrophil-to-lymphocyte ratio (NLR) was calculated by diving the neutrophil count by the lymphocyte count. The lymph node ratio (LNR) was calculated by dividing the positive lymph node value by the total number of examined nodes. Other clinicopathological data of GI-PDNEN patients, including gender, age, tumor location, tumor size, Ki-67 index, TNM stage, and adjuvant therapy, were retrospectively collected. Location of the primary tumor was classified as foregut (esophagus, stomach, and proximal duodenum; excluding pancreas), midgut (distal duodenum, appendix, and proximal colon), and hindgut (colon and rectum).

### Patient Follow-Up

Postoperative follow-up was regularly conducted for every patient through outpatient visits, telephone calls, letters, or the Internet. The investigations items included clinical symptoms, detection of biochemical indexes, routine imaging (CT / MRI) and endoscopy. Dates of follow-up and recurrence, and vital status data were collected. The last follow-up in the present study was carried out in January 2020. The primary endpoint of this study was early recurrence, disease-free survival (DFS), and overall survival (OS). DFS was defined as the time from surgery to relapse, death, or last follow-up. OS was defined as the time from surgery to death from any cause or last follow-up.

### Statistical Analysis

Categorical variables were expressed as frequency and percentage, while continuous variables were presented as mean ± standard deviation or median with interquartile region (IQR). Categorical variables were compared using Pearson's chi-square test or Fisher's exact test. Continuous variables were compared using Student's *t*-test or the Mann-Whitney *U*-test. The cutoff values for PLR, NLR, ALP, and LNR on early recurrence were determined using receiver operating curve (ROC) analysis. The Kaplan-Meier method was used to calculate the cumulative survival rate and generate survival curves, which were compared using the log-rank test. Differences in the clinicopathological characteristics of patients between the early recurrence and non-early recurrence groups were investigated. Risk factors for early recurrence after surgery were identified by univariate and multivariate logistic regression analyses using the forward stepwise (likelihood ratio) method. The impact of various clinicopathological factors on early recurrence was assessed through odds ratio (OR) and 95% confidence interval (CI). A two-tailed *p-*value of < 0.05 was considered to indicate statistical significance. All statistical analyses were performed using SPSS version 25.0 for Windows (IBM, Armonk, New York, USA).

## Results

### Demographic Characteristics

**Figure 1:**
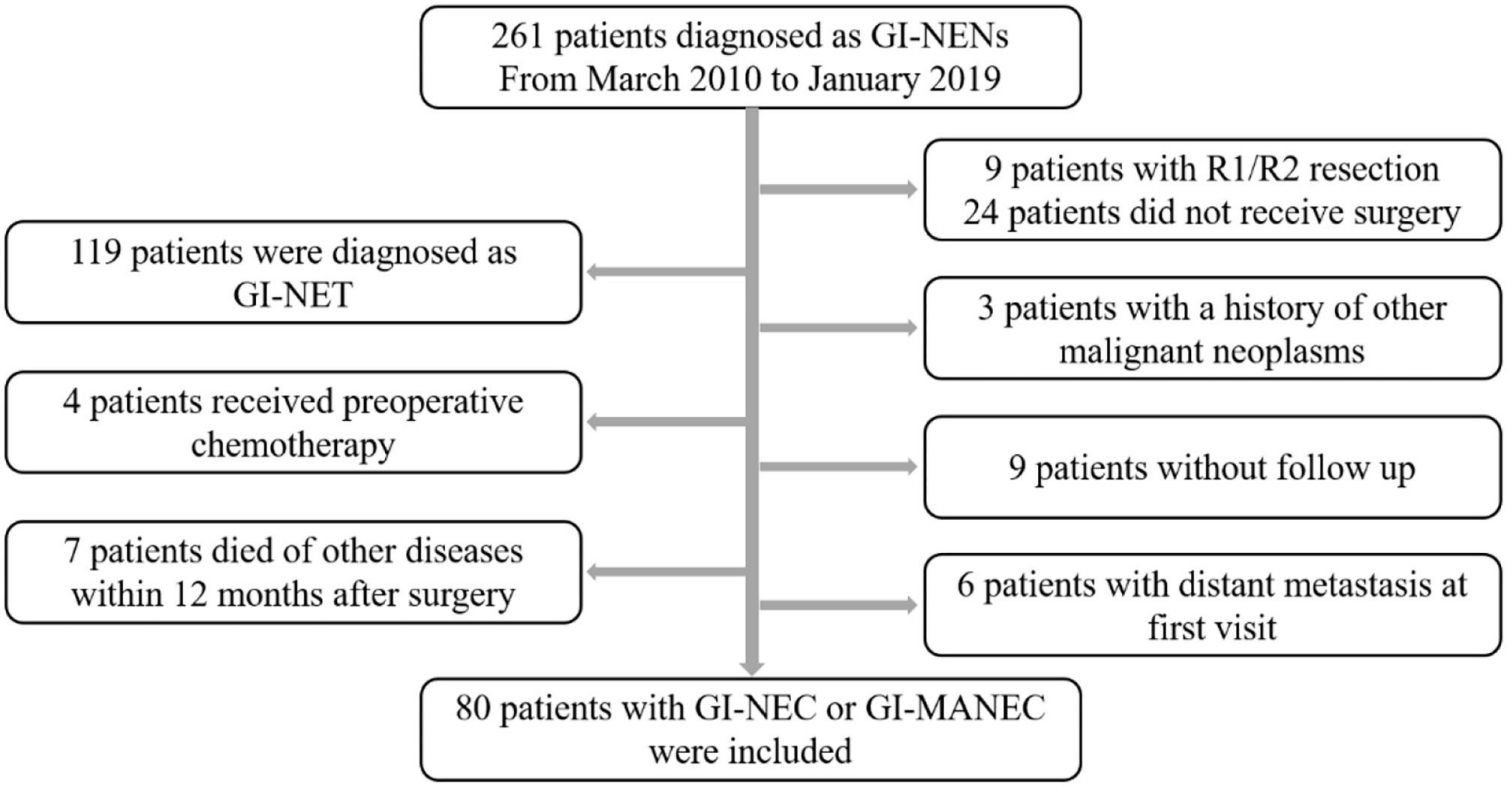
Study scenario of the patients undergoing surgical resection for GI-PDNEN.

A total of 80 patients were included in this study (Figure 1), of which 55 patients were male (68.8%) and 25 were female (30.2%). The median patient age was 60 years (range 38–85). None of the patients had clinical symptoms related to hormones. Most of the tumors were located in the foregut (*n* = 66, 82.5%), followed by hindgut (*n* = 9, 11.2%), and 5 (6.2%) were located in the midgut. The mean tumor size was 4.69 ± 2.70 cm. Among the 80 patients, 52 (65.0%) were diagnosed with GI-NEC and 28 (35.0%) with GI-MANEC. According to AJCC staging, five cases (6.3%) were stage I, 10 (12.5%) were stage II, and 65 (81.3%) were stage III. Forty-one patients received adjuvant chemotherapy. Thirty-five patients received platinum-containing regimens including etoposide plus cisplatin (EP) or fluorouracil/leucovorin/oxaliplatin combinations (FOLFOX), while the remaining six patients received a platinum-free regimen. Because all patients in this study had non-functional tumors, none of the patients received treatment with somatostatin analogs after surgery. The demographic characteristics of the patients are shown in Table 1 and Supplementary Table 1.

**Table 1:** Demographic characteristics of all the patients.

	**All patients**	**Early recurrence**	
		**No (%)**	**Yes (%)**
**Sex**			
Male	55 (68.8%)	35 (66.0%)	20 (74.1%)
Female	25 (31.2%)	18 (34.0%)	7 (25.9%)
**Age (years)**			
<60	43 (53.8%)	27 (50.9%)	16 (59.3%)
≥60	37 (46.2%)	26 (49.1%)	11 (40.7%)
**Location**			
Foregut	66 (82.5%)	49 (92.5%)	17 (63.0%)
Midgut	5 (6.2%)	3 (5.7%)	2 (7.4%)
Hindgut	9 (11.2%)	1 (1.9%)	8 (29.6%)
**Preoperative factors**			
**PLR**			
≤ 174	59 (73.8%)	43 (81.1%)	16 (59.3%)
>174	21 (26.2%)	10 (18.9%)	11 (40.7%)
**NLR**			
≤ 2.26	41 (51.2%)	31 (58.5%)	10 (37.0%)
>2.26	39 (48.8%)	22 (41.5%)	17 (63.0%)
**ALP**			
≤ 80	55 (68.8%)	42 (79.2%)	13 (48.1%)
>80	25 (31.2%)	11 (20.8%)	14 (51.9%)
Tumor size (cm)	4.7 ± 2.7	4.8 ± 2.7	4.5 ± 2.7
**WHO 2010**			
NEC	52 (65.0%)	32 (60.4%)	20 (74.1%)
MANEC	28 (35.0%)	21 (39.6%)	7 (25.9%)
**pT stage**			
T1-T2	16 (20.0%)	13 (24.5%)	3 (11.1%)
T3-T4	64 (80.0%)	40 (75.5%)	24 (88.9%)
**Lymph node metastasis**			
No	25 (31.2%)	20 (37.7%)	5 (18.5%)
Yes	55 (68.8%)	33 (62.3%)	22 (81.5%)
**LNR**			
≤ 0.25	54 (67.5%)	44 (83.0%)	10 (37.0%)
>0.25	26 (32.5%)	9 (17.0%)	17 (63.0%)
**TNM stage**			
I-II	18 (22.5%)	16 (30.2%)	2 (7.4%)
III	62 (77.5%)	37 (69.8%)	25 (92.6%)
**Ki-67**			
≥80%	46 (57.5%)	30 (56.6%)	16 (59.3%)
<80%	34 (42.5%)	23 (43.4%)	11 (40.7%)
**Adjuvant chemotherapy**			
No	39 (48.8%)	26 (49.1%)	13 (48.1%)
Yes	41 (51.2%)	27 (50.9%)	14 (51.9%)
**Adjuvant chemotherapy regimens**			
No	39 (48.8%)	26 (49.1%)	13 (48.1%)
Platinum-containing regimens	35 (43.8%)	26 (49.1%)	9 (33.3%)
Other regimens	6 (7.5%)	1 (1.9%)	5 (18.5%)

### Follow-Up Results and Survival Analysis

At the last follow-up, the median survival time of the whole cohort was 46 months (range 5–85). During the follow-up period, 44 patients (55.0%) had disease recurrence, of which early recurrence occurred in 27 patients, while the other 17 patients developed late recurrence. Among the whole cohort, the most common site of recurrence was the liver (*n* = 35), followed by local lymph nodes (*n* = 4), and three patients had metastases of other sites including the lung, bone, and brain. During follow-up, 35 patients died, of which 30 died due to GI-PDNEN and the remaining five died due to other causes such as cardiovascular and cerebrovascular diseases or accidents.

The 1- and 3-year DFS rates of the whole cohort were 61.1 and 39.8%, respectively, and the one- and 3-year OS rates were 79.8 and 52.0%, respectively. The median OS of patients in the non-early recurrence group was not reached, while that of the patients in the early recurrence group was 12 months (*p* < 0.001). The OS of patients with no recurrence was superior to that of patients with early recurrence or recurrence after 12 months (*p* < 0.001) (Supplementary Figure 1).

### Univariate and Multivariate Analyses of Postoperative Early Recurrence in Patients With GI-PDNEN

The median values of PLR, NLR, ALP, and LNR in the whole cohort were 139 (IQR, 120–177), 2.29 (IQR, 1.73–3.32), 69 (IQR, 60–88), and 0.13 (IQR, 0–0.32), respectively. According to ROC analysis, the optimal cutoff values of PLR, NLR, ALP, and LNR for early recurrence were 174, 2.26, 80, and 0.25, respectively. All factors were divided into two variables according to category, cutoff values, and mean or median values. Univariate analysis revealed that tumor location, PLR, preoperative ALP, LNR, and TNM stage are related to early recurrence of GI-PDNEN (*p* < 0.05) (Table 2). Including these four factors in the multivariate analysis, using the forward stepwise (likelihood ratio) method, showed that tumor location, ALP, and LNR were independent factors influencing postoperative early recurrence in patients with GI-PDNEN (Table 3). Patients with tumors located in the midgut/hindgut (β = 1.625; OR = 5.077, 95% CI 1.058–24.352, *p* = 0.042), ALP >80 (β = 1.674; OR = 5.331, 95% CI 1.557–18.258, *p* = 0.008), and LNR >0.25 (β = 1.884; OR = 6.578, 95% CI 1.971–21.951, *p* = 0.002) were associated with a higher risk of postoperative early recurrence. In the multivariate analysis, the risk of early recurrence for patients with GI-PDNENs was


$$\begin{array}{l} P=\\ \frac{\text{exp}(1.625\times \text{location}+1.674\times \text{ALP}+1.884\times \text{LNR}-3.892)}{1+\text{exp}(1.625\times \text{location}+1.674\times \text{ALP}+1.884\times \text{LNR}-3.892)}. \end{array}$$

**Table 2:** Univariate analysis of the risk factors associated with early recurrence for GI-PDNEN.

	**OR (95% CI)**	***P-*value**
Sex (female)	0.681 (0.243–1.909)	0.465
Age (>60 years)	0.714 (0.280–1.824)	0.481
Location (midgut/hindgut)	7.206 (1.995–26.023)	0.003
PLR (>174)	2.956 (1.054–8.288)	0.039
NLR (>2.26)	2.395 (0.923–6.214)	0.072
ALP (>80)	4.112 (1.505–11.236)	0.006
WHO2010 (MANEC)	0.533 (0.192–1.482)	0.228
Tumor size (>5)	0.695 (0.257–1.880)	0.473
pT stage (T3-4)	2.600 (0.672–10.065)	0.166
Lymph node metastasis	2.667 (0.871–8.162)	0.086
LNR (>0.25)	8.311 (2.879–23.996)	<0.001
TNM stage (III)	5.405 (1.141–25.597)	0.033
Ki-67 (≥80%)	0.897 (0.250–2.297)	0.82
Adjuvant chemotherapy	1.037 (0.410–2.621)	0.939

**Table 3:** Multivariate analysis of the risk factors associated with early recurrence for GI-PDNEN.

	**β**	**Wald**	**OR**	***P-*value**
Location (midgut/hindgut)	1.625	4.124	5.077 (1.058–24.352)	0.042
ALP (>80)	1.674	7.1	5.331 (1.557–18.258)	0.008
LNR (>0.25)	1.884	9.388	6.578 (1.971–21.951)	0.002

### Establishment and Validation of a Risk Predictive Scoring Model for Early Recurrence

**Figure 2:**
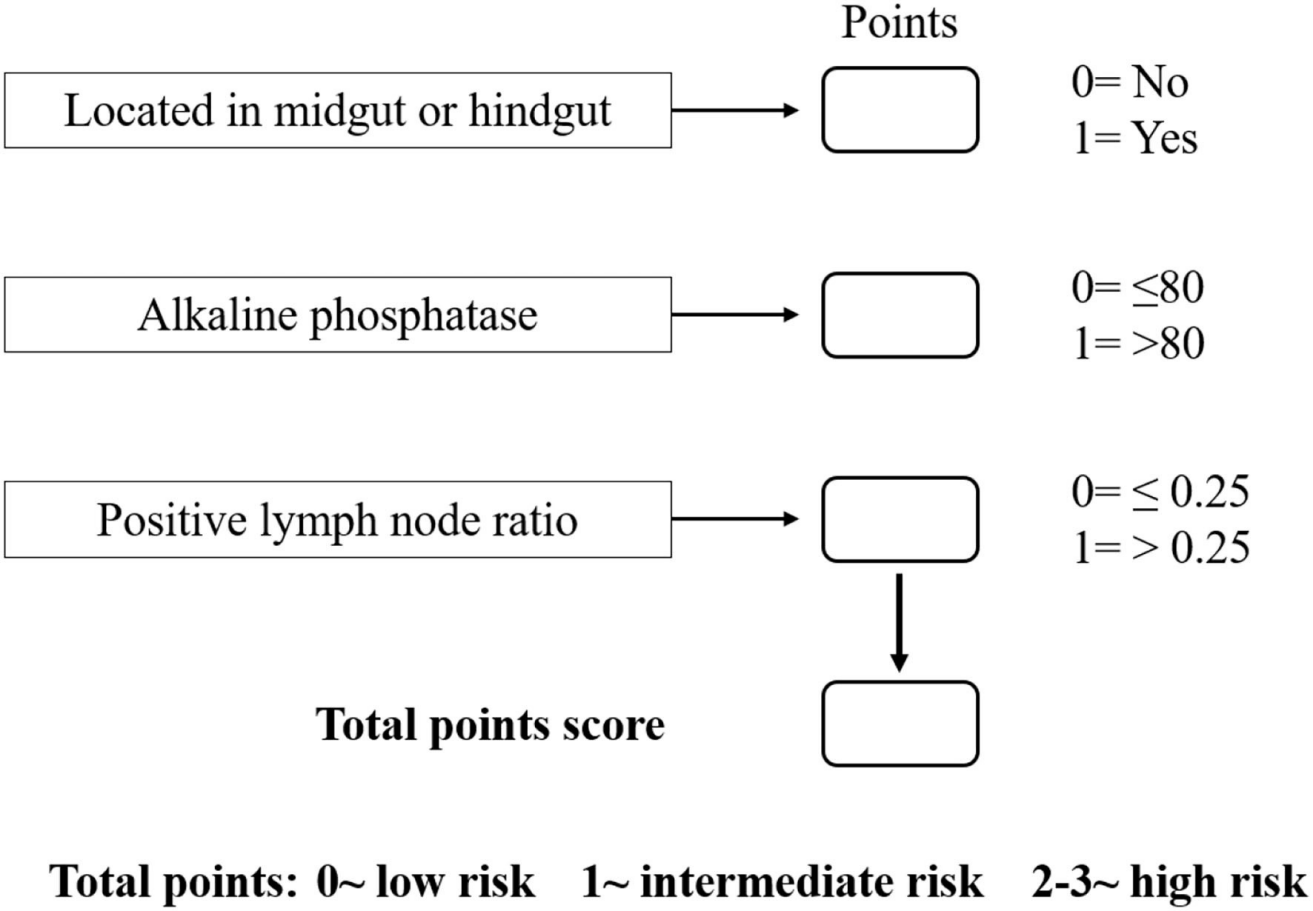
Risk scoring model for early recurrence of GI-PDNEN.

**Figure 3:**
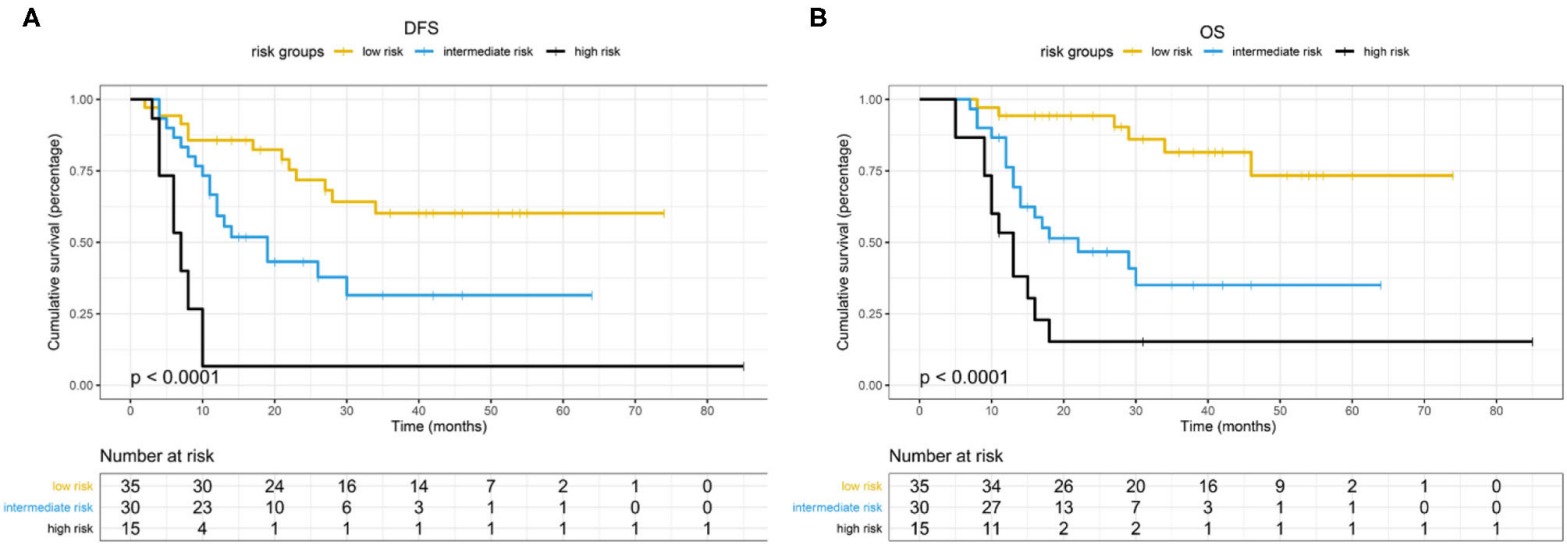
Kaplan-Meier analyses of different risk groups of GI-PDNEN. **(A)** DFS. **(B)** OS.

Based on the β values of the three aforementioned factors identified in multivariate analysis, a risk predictive scoring model for early recurrence was established (Figure 2). The ratio of the β values of location, ALP, and LNR was 0.863, 0.889, and 1.000 respectively. For the convenience of clinical application, the risk scores were assigned to tumor location (foregut, 0; midgut/hindgut, 1), ALP (≤80, 0; >80, 1), and LNR (≤0.25, 0; >0.25, 1). According to the total points scored, patients with GI-PDNEN were stratified into three groups: 0 point as low risk of postoperative early recurrence, 1 point as intermediate risk, and ≥2 points as high risk. The recurrence rate within 12 months after surgery in the high-risk group was significantly higher than that in the intermediate- and low-risk groups (*p* < 0.001) (Table 4). To validate the prediction efficiency of this model, a comparison with the TNM stage on early recurrence was conducted. The corresponding ROC analysis showed that the AUC of the prediction scoring model was significantly higher than that of the TNM stage (0.795 vs. 0.614, *p* = 0.003). Additionally, the DFS and OS of patients with low risk were significantly superior to those of intermediate- or high-risk groups (Figure 3). The 1- and 3-year DFS and OS rates of patients according to risk groups are shown in Table 4.

**Table 4:** Recurrence patterns and 1-, 3- DFS/OS of different groups.

	**Risk of early recurrence**			***P-*value**
	**Low**	**Intermediate**	**High**	
Early recurrence				<0.001
Yes	5	8	14	
No	30	22	1	
Adjuvant chemotherapy				<0.001
Early recurrence	2	3	9	
No early recurrence	12	14	1	
Recurrence patterns				
Liver	5	6	11	
Local recurrence	0	1	1	
Other locations	0	1	2	
DFS				<0.001
1-year	85.7%	59.3%	6.7%	
3-year	60.2%	31.5%	6.7%	
OS				<0.001
1-year	94.3%	86.7%	53.3%	
3-year	81.5%	35.0%	15.2%	

## Discussion

It has been well-documented that early recurrence after surgery leads to dismal prognosis (10–12). Because there is no consensus on the optimal threshold for differentiating early and late recurrence of GI-PDNEN, early recurrence was defined as recurrence within the 1st year after surgery, which is in line with that used in previous studies (10–12). With a relatively large sample size from one single-center institution in China, we demonstrated that the rate of early recurrence after curative surgery in GI-PDNEN was 30.7% and the median overall survival was 12 months. We also investigated the risk of early recurrence after curative surgery and scored this risk according to preoperative and postoperative clinicopathological factors.

Knowledge about the factors influencing early recurrence of GI-PDNEN remains scarce. Preoperative inflammatory and biochemical markers such as PLR, NLR, and ALP have been reported to play an important role in the prognosis of patients with GI-PDNEN (13–15). In the present study, we analyzed the relationship between these factors and early recurrence and found that preoperative ALP is a more meaningful indicator to predict the early recurrence of GI-PDNEN than inflammatory makers. GI-PDNEN patients with preoperative ALP >80 are at an increased risk of early recurrence. Similarly, Lamarca et al. elaborated that preoperative ALP ≥83 is a risk factor for the overall survival of GI-NEC patients (14). ALP is an enzyme that can dephosphorylate multiple substrates. Serum ALP is mainly derived from the liver and bone tissue. Therefore, in patients with GI-PDNENs with elevated levels of serum ALP, attention should be paid to the risk of metastasis to liver or bone. In addition, our study also demonstrated that an increased LNR, rather than lymph node metastasis (LNM), is related to an increased risk of early recurrence, which means that the risk of early recurrence in some patients with lymph node metastasis may be overestimated. Therefore, the determination of the LNR may, to some extent, decrease the possibility of risk migration compared with lymph node metastasis. Nevertheless, because the value of the LNR is related to the total number of lymph nodes dissected, this finding should be carefully analyzed and further studies are required to verify our results. Additionally, tumor location was also an independent factor for the early recurrence of GI-PDNEN in this study.

Predictive models, which may provide a personalized assessment of the prognosis using patient-specific characteristics, have been increasingly incorporated into clinical practice in the field of GI-NEN (16–18). In this study, with the aforementioned three independent risk factors, we established a scoring model to predict the risk of postoperative early recurrence in GI-PDNEN patients. To the best of our knowledge, this is the first predictive scoring model for early recurrence of GI-PDNEN. According to the total score points, patients with GI-PDNEN were stratified into three groups for the risk of early recurrence: low risk, intermediate risk, and high risk. The risk of postoperative early recurrence in the high-risk group (score, 2–3) was significantly higher than that in the low-risk (score, 0) or intermediate-risk (score, 1) groups, which indicates that intensive follow-up and active adjuvant therapy may be required for these patients. Furthermore, we compared the DFS and OS of the three groups of patients, and the scoring model also showed a good stratification for the prognosis of GI-PDNEN patients. Additionally, the novel scoring model has a superior predictive efficiency for early recurrence over TNM staging according to ROC analysis. Because it is a convenient, cost-effective, and reliable model, the novel scoring model may assist in clinical treatment and postoperative follow-up. Furthermore, it may also influence clinical trial design with respect to patient stratification.

Currently, adjuvant chemotherapy of GI-PDNEN patients remains controversial. Platinum-containing chemotherapy regimens, especially the etoposide and cisplatin (EP) regimen, are the most commonly used (19). Given the heterogeneous nature of GI-PDNEN, the efficacy of adjuvant chemotherapy varies. A few studies with small sample sizes have reported that for esophageal or gastric PDNEN patients, improved survival was observed for patients receiving adjuvant chemotherapy after surgery as compared to surgery alone (6, 20). However, adjuvant chemotherapy did not benefit the three groups of patients in this study. The difference may be owing to the small number of patients receiving each chemotherapy regimen, which might result in bias. In addition, the patients in this study received different adjuvant treatments, which may also weaken the influence of adjuvant therapy on the prognosis. Therefore, the optimal adjuvant chemotherapy regimen for GI-PDNEN still requires further research.

There are some limitations in this study. First, this study was a single-center retrospective study; hence, there might be an intrinsic selection bias. Additionally, although the time span of this study was as long as 9 years, the sample size was relatively small, which may be due to the low incidence of GI-PDNEN and may have decreased the robustness of the study. The definition of early recurrence in GI-PDNEN patients is still not very clear and external validation was not performed in our study, further multicenter studies with large sample sizes are still required to confirm the findings. Finally, due to the high heterogeneity of GI-PDNEN, its prognosis mainly depends on its biological behavior. In-depth understanding of its molecular mechanism is of great significance for improving the prognosis of patients, and this is also the direction that needs to be studied in the future.

## Conclusion

In conclusion, tumor location, preoperative ALP, and LNR are independent factors influencing early recurrence of GI-PDNENs. The novel scoring model has superior predictive efficiency for early recurrence of GI-PDNENs and may assist in clinical treatment.

## Data Availability Statement

The raw data supporting the conclusions of this article will be made available by the authors, without undue reservation.

## Ethics Statement

This retrospective study was approved by the Ethics Committee of Union Hospital, Tongji Medical School, Huazhong University of Science and Technology (no. 2021-0181). Informed consent was obtained from all patients included in this study.

## Author Contributions

KT contributed to the conception and administrative support of the study. CheL and PZ contributed to the study design. XS and WL contributed to the collection and assembly of the data. PZ and WL contributed to the quality control of data. XT, XC, and WY contributed to the data analysis and interpretation. CheL and ChoL prepared the manuscript. ZW and KT edited the manuscript. All authors reviewed the results and approved the final version of the manuscript.

## Funding

This study was supported by National Natural Science Foundation of China (Grant Nos. 81702386 and 81874184) and the Key project of Hubei health commission (Grant No. WJ2019Q030).

## Conflict of Interest

The authors declare that the research was conducted in the absence of any commercial or financial relationships that could be construed as a potential conflict of interest.

## Publisher's Note

All claims expressed in this article are solely those of the authors and do not necessarily represent those of their affiliated organizations, or those of the publisher, the editors and the reviewers. Any product that may be evaluated in this article, or claim that may be made by its manufacturer, is not guaranteed or endorsed by the publisher.

## Abbreviations

NENs: Neuroendocrine neoplasms
GI-PDNEN: Gastrointestinal neuroendocrine carcinoma and mixed adenoneuroendocrine carcinoma
WHO: World Health Organization
NET: Neuroendocrine tumor
NEC: neuroendocrine carcinoma
MANEC: mixed adenoneuroendocrine carcinoma
HPF: High-power fields
AJCC: American Joint Committee on Cancer
ALP: Alkaline phosphatase
PLR: Platelet-to-lymphocyte ratio
NLR: Neutrophil-to-lymphocyte ratio
LNR: The lymph node ratio
DFS: Disease-free survival
OS: Overall survival
IQR: Interquartile region
ROC: Receiver operating curve
OR: Odds ratio
CI: Confidence interval
EP: Etoposide plus cisplatin
FOLFOX: fluorouracil /leucovorin/oxaliplatin combinations.
