# Supplementary material for: Risk Factors and Predictive Score Model for Early Recurrence After Curative Surgery in Patients With Poorly Differentiated Gastrointestinal Neuroendocrine Neoplasms
Source: Front Surg. 2021 Sep 16;8:703138. doi: 10.3389/fsurg.2021.703138 (PMC8481802; doi:10.3389/fsurg.2021.703138)
Supplement: Supplementary file 1 [file Data_Sheet_1.pdf]

Supplementary Table 1 The demographic characteristics of the patients with early recurrence, late recurrence (postoperative recurrence after 12 months), and no recurrence

	Early recurrence (%)	Late recurrence (%)	No recurrence (%)	*P* value
Sex				0.564
male	20 (74.1%)	10 (58.8%)	25 (69.4%)	
female	7 (25.9%)	7 (41.2%)	11 (30.6%)	
Age (years)				0.57
<60	16 (59.3%)	10 (58.8%)	17 (47.2%)	
≥60	11 (40.7%)	7 (41.2%)	19 (52.8%)	
Location				0.002
foregut	17 (63.0%)	15 (88.2%)	34 (94.4%)	
midgut	2 (7.4%)	2 (11.8%)	1 (2.8%)	
hindgut	8 (29.6%)	0 (0.0%)	1 (2.8%)	
Preoperative factors				
PLR				0.036
⩽174	16 (59.3%)	16 (94.1%)	27 (75.0%)	
>174	11 (40.7%)	1 (5.9%)	9 (25.0%)	
NLR				0.159
⩽2.26	10 (37.0%)	11 (64.7%)	20 (55.6%)	
>2.26	17 (63.0%)	6 (35.3%)	16 (44.4%)	
ALP				0.017
⩽80	13 (48.1%)	14 (82.4%)	28 (77.8%)	
>80	14 (51.9%)	3 (17.6%)	8 (22.2%)	
Tumor size (cm)	4.5±2.7	4.1±1.9	5.1±3.0	0.421
WHO 2010				0.353
NEC	20 (74.1%)	9 (52.9%)	23 (63.9%)	
MANEC	7 (25.9%)	8 (47.1%)	13 (36.1%)	
pT stage				0.388
T1-T2	3 (11.1%)	4 (23.5%)	9 (25.0%)	
T3-T4	24 (88.9%)	13 (76.5%)	27 (75.0%)	
Lymph node metastasis				0.02
Yes	5 (18.5%)	3 (17.6%)	17 (47.2%)	
No	22 (81.5%)	14 (82.4%)	19 (52.8%)	
LNR				<0.001
⩽0.25	10 (37.0%)	12 (70.6%)	32 (88.9%)	
>0.25	17 (63.0%)	5 (29.4%)	4 (11.1%)	
TNM stage				0.023
I-II	2 (7.4%)	3 (17.6%)	13 (36.1%)	
III	25 (92.6%)	14 (82.4%)	23 (63.9%)	
Ki-67				0.358
≥80%	16 (59.3%)	12 (70.6%)	18 (50.0%)	
<80%	11 (40.7%)	5 (29.4%)	18 (50.0%)	
Adjuvant chemotherapy				0.386

No	13 (48.1%)	6 (35.3%)	20 (55.6%)	
Yes	14 (51.9%)	11 (64.7%)	16 (44.4%)	
Adjuvant chemotherapy regimens				0.044
No	13 (48.1%)	6 (35.3%)	20 (55.6%)	
Platinum-containing regimens	9 (33.3%)	10 (58.8%)	16 (44.4%)	
Other regimens	5 (18.5%)	1 (5.9%)	0 (0.0%)	

Supplementary Fig 1 Kaplan-Meier analyses of GI-PDNEN patients with early recurrence, late recurrence (postoperative recurrence after 12 months), and no recurrence

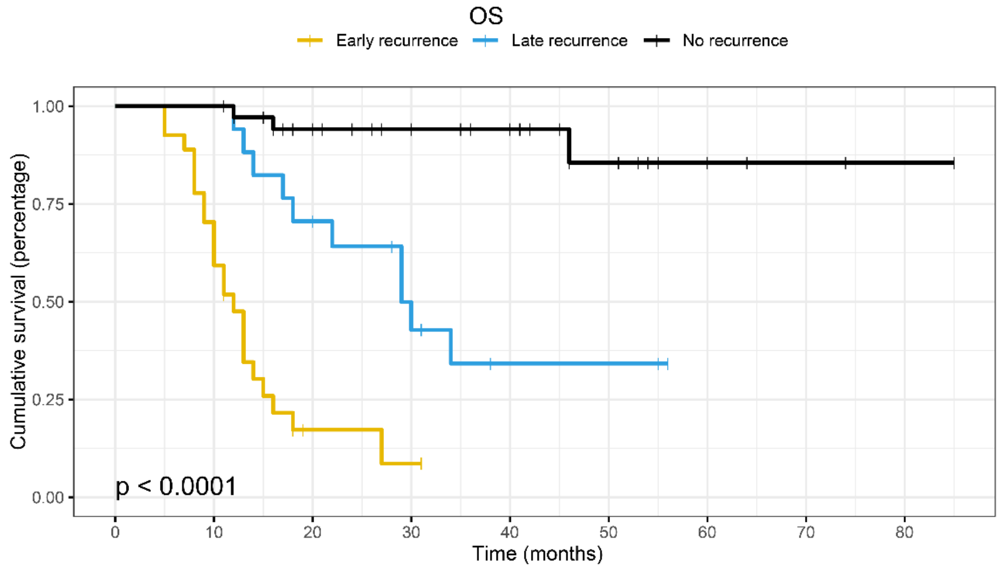

Number at risk

Early recurrence	27	19	2	1	0	0	0	0	0
Late recurrence	17	17	12	7	2	2	0	0	0
No recurrence	36	36	27	21	18	9	4	2	1
